# Epidemiology, Pathogenesis, and Treatment Options of Monkeypox: A Narrative Review

**DOI:** 10.7759/cureus.77892

**Published:** 2025-01-23

**Authors:** Priyavardhan Mishra, Ratnav Singh, Anant Patil

**Affiliations:** 1 Medicine, DY Patil Deemed to be University, School of Medicine, Mumbai, IND; 2 Department of Pharmacology, Dr. Reddy’s Laboratories, Mumbai, IND; 3 Pharmacology, DY Patil Deemed to be University, School of Medicine, Mumbai, IND

**Keywords:** antiviral therapy, monkeypox, mpox, orthopoxvirus, tecovirimat, zoonotic infection

## Abstract

Recently, human mpox (monkeypox) has emerged as a global outbreak. This debilitating disease is the result of a zoonotic infection and often entails multiorgan involvement. The knowledge of pathophysiology and treatment options is evolving. This review aims to summarise the pathophysiology and current treatment options of mpox. Prevention and vaccination strategies are out of the scope of this review. We performed a literature review to achieve this objective. Mpox is a zoonotic infection caused by the monkeypox virus, a double-stranded DNA virus. The virus, with debilitating complications, affects multiorgan functions, including skin and other systems. The available treatments for this widespread infection are limited. Only a few antivirals have been sanctioned and approved by the regulatory authorities. We have summarised the efficacy and safety of the three antiviral agents, namely, cidofovir (CDV), brincidofovir (BDV), and tecovirimat. The available limited evidence points towards promising efficacy and tolerability of tecovirimat.

## Introduction and background

Monkeypox history and epidemiology

Monkeypox (mpox) refers to a zoonotic disease caused by the monkeypox virus (MPXV), a double-stranded DNA virus of the *Orthopoxvirus* genus within the Poxviridae family and Chordopoxvirinae subfamily [1]. MPXV is classified into clades I and II based on geographic occurrence; clade II is further subclassified into clades IIa and IIb [2]. The first case of mpox in humans was reported in a nine-year-old child at the Basankusu Hospital in the Democratic Republic of Congo on September 1, 1970 [1,3,4]. However, in 1958, mpox was first recorded as an outbreak in monkeys at a research institute in Copenhagen, Denmark [1,3,4]. Afterwards, between October 1970 and May 1971, six human cases of mpox were recorded in Liberia, Nigeria, and Sierra Leone [1,3]. In Nigeria, the first index case of mpox was documented in 1971, followed by 10 more cases between 1971 and 1978 [3,5]. Subsequently, several thousands of human mpox cases were confirmed in 15 countries, out of which 11 were in Africa [1,3]. 

Subsequently, mpox was declared endemic in multiple African nations [1,6,7]. In 2003, the first non-endemic mpox outbreak occurred in the USA from rodents imported from Ghana [1,8-10]. Between 2018 and 2021, 11 cases were reported in the United Kingdom, USA, and Singapore, caused by cross-border tourism [1]. A total of 88,600 cases of mpox and 152 deaths, representing a case fatality rate of 0.17%, have been recorded in 113 countries, of which 106 had no record of the disease as of August 2, 2023 [1,11]. Since the commencement of mpox monitoring by the World Health Organization (WHO) in 2022, as of July 31, 2024, a total of 102,977 confirmed cases of the disease and 219 fatalities have been reported from 121 countries [12]. In 2024, over 20,000 mpox cases have been reported from 13 African countries, with over 3000 confirmed cases and over 500 deaths (case fatality rate of 2.9%) from 13 African countries [12]. A resurgence of mpox as a ‘public health emergency of international concern’ (PHEIC) was declared by the WHO on August 14, 2024 [13]. 

Mpox in India

On July 14, 2022, India reported its first case of mpox, from the southern state of Kerala; there have been 30 reported cases since the PHEIC announcement [14,15]. On August 12, 2024, the National Centre for Disease Control (NCDC) summoned an expert gathering to analyze the threat of mpox [15]. The NCDC updated its prior Communicable Disease alert on mpox to reflect new developments [15]. On August 16, 2024, a Joint Monitoring Group Meeting including members from the NCDC, WHO, Indian Council of Medical Research, National Centre for Vector Borne Disease Control Programme, Directorate General of Health Services (DGHS), Central Government Hospitals, and All India Institute of Medical Sciences was held under the chairmanship of the DGHS [15]. It was also mandated that the network of testing laboratories be updated to ensure early detection [15]. The objective of the review was to compile evidence regarding the epidemiology, pathogenesis, and treatment options of mpox. 

## Review

Methods

We searched PubMed/MEDLINE and Google Scholar databases for relevant articles published until September 2024 using the following keywords: “Monkeypox”, “MPOX”, “Cidofovir”, "Brincidofovir” and “Tecovirimat”. Detailed evidence on treatment options was also searched through regulatory websites such as the United States Food and Drug Administration (USFDA) and the European Medicine Agency (EMA). We included all articles with the above keywords in the title and/or abstract. Articles published in languages other than English were excluded. Prevention and vaccination options against mpox were not in the scope of our review.

Pathogenesis and clinical features

Transmission of mpox occurs either through zoonoses or from human sources [16]. Zoonotic dissemination occurs through direct contact with diseased parts, bodily fluids, or contaminated objects, scratching or biting of infected animals, and consumption of contaminated animal flesh [16]. Mpox is transmitted from a human source by close contact with an individual infected with MPXV, including contact with respiratory secretions, skin lesions, genitalia, contaminated bedding and clothing [16]. The outcomes of mpox on pregnancy are less documented; however, there is evidence of vertical transmission of MPXV in the literature [16-18]. Following transmission, the incubation period of MPXV varies between five and 21 days (usually seven to 14 days); subsequently, symptoms appear and the disease manifests for two to five weeks [5,19,20].

**Figure 1:**
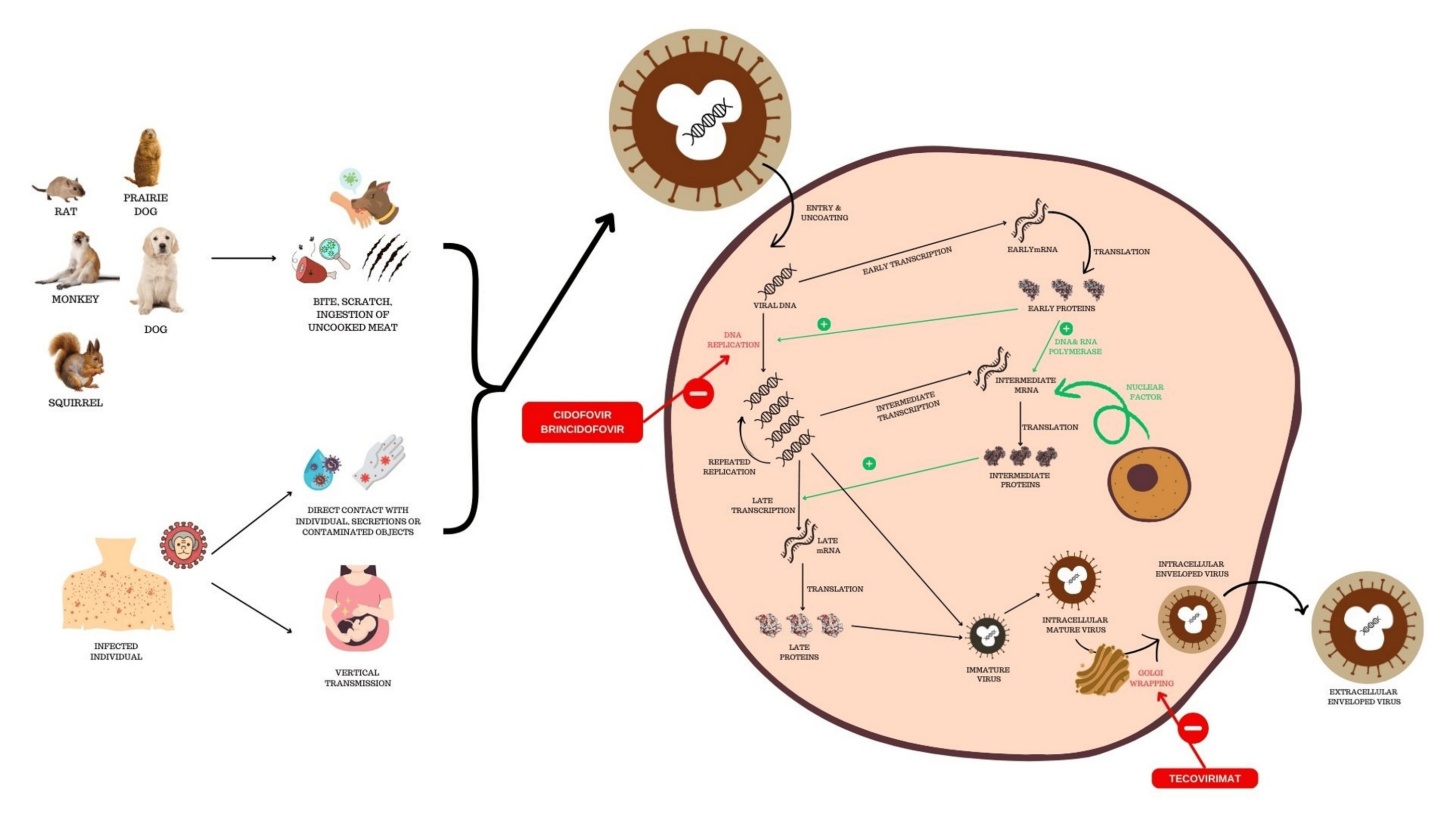
Transmission and pathogenesis of mpox along with the mechanism of action of tecovirimat, cidofovir, and brincidofovir The virus is transmitted from animal sources, resulting in multiplication inside the human cell. Cidofovir and brincidofovir inhibit viral DNA replication. Tecovirimat acts by inhibiting the envelope-wrapping protein. The figure is an original image created by the authors.

**Figure 2:**
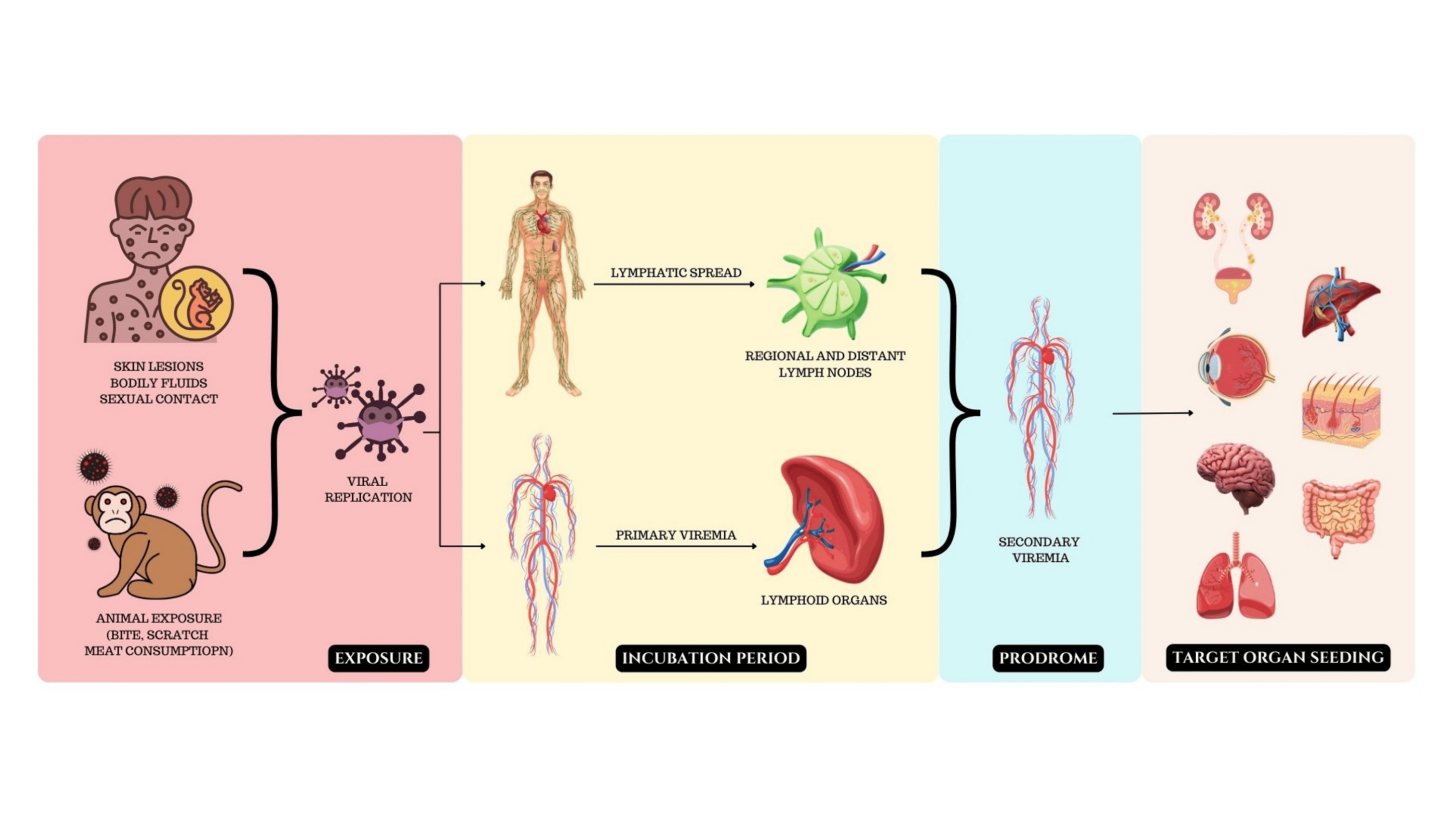
Spread of infection and multi-organ involvement in mpox. The virus spreads through lymphatics and blood, resulting in prodromal symptoms. Later, the virus affects multiple organs including the liver, kidney, eye, intestine, lung, brain and skin. The figure is an original image created by the authors.

MPXV attacks the cutaneous membranes, soft tissues, and upper or lower respiratory tract mucosa activating a local innate immune response that leads to the recruitment of macrophages, fibroblasts, and polymorphonuclear leukocytes [5,19]. The virus evades the host’s defence system and is carried via the lymphatic system to regional lymph nodes, which leads to a primary viremic phase with seeding in the spleen and tonsils [19,21]. Seeding in the integuments and organs (including the liver, intestines, kidneys, ovaries, testicles, and brain) occurs during the secondary viremic phase [19,21]. Clade I type of MPVX causes a higher number of cutaneous lesions, greater involvement of the gastrointestinal tract by producing granuloma, and prolongs the duration of infection with more severe clinical features and a 10-fold higher plasma viral load when compared with infection caused by clade II [19]. The detailed pathogenesis is illustrated in Figures 1-2.

Mpox begins with nonspecific disease manifestations following the incubation period [19-21]. The early nonspecific signs and symptoms of the disease are listed in Table 1 [19-21]. After one to five days of the onset of pyrexia, multiple rashes of various sizes appear starting from the face, progressing to the torso, and subsequently appearing in the upper and lower limbs [19,20]. Around the discrete skin lesions, areas of erythema and hyperpigmentation are often noted [20]. The lesions are typically larger and more numerous in patients who have not received smallpox vaccination when compared with vaccinated patients [19]. The most common post-viral sequelae of human mpox infection are secondary bacterial infections of the skin manifesting as cellulitis and carbuncles and leading to permanent skin disfiguration, alopecia, or pigmentation changes in unvaccinated or immunocompromised individuals [19]. Mpox also affects multiple systems including the respiratory, gastrointestinal, ophthalmic, genitourinary, and central nervous systems, ophthalmic and genitourinary [19].

**Table 1:** Clinical features and manifestations of mpox References: [19-22]

Non-specific signs and symptoms	
Prodrome phase	Fever, chills, headache, hyperhidrosis (excessive sweating), asthenia (generalized weakness/fatigue), backache, malaise, lethargy, myalgia (muscle weakness), lymphadenopathy of the neck, axilla and/or inguinal nodes, sore throat, cough (productive or non-productive).
Systemic manifestations	
Skin manifestations	Polymorphic centrifugal rash, Evolution from macules, papules, vesicles, and pustules to resolution with crusts and scab. Size of lesions: 0.5 to 1 cm in diameter, mild; ≤25 lesions, moderate; 25–99 lesions, severe; 100–250 lesions, serious; > 250 lesions
Oral manifestations	Pharyngitis, oral ulcers, tonsillitis
Gastrointestinal manifestations	Nausea, vomiting, watery diarrhea, dehydration, malnutrition, hepatitis
Central nervous system manifestations	Encephalitis, meningoencephalitis, seizures
Ophthalmic manifestations	Conjunctivitis, blepharitis, blepharoconjunctivitis, keratitis, subconjunctival nodules, corneal ulcers, photophobia
Genitourinary manifestations	Lesions on external genitalia, penile edema, regional tender lymphadenopathy, dysuria

Treatment options

With no specific treatment available for mpox, supportive measures and antivirals with activity against MPXV continue to be the mainstay to tackle the disease [19,20,22]. Three antivirals, namely, tecovirimat, cidofovir (CDV), and brincidofovir (BDV), are the available treatment options against mpox; however, their efficacy is yet to be evaluated in randomized or non-randomized trials [22]. These drugs are currently approved for the management of smallpox based on results derived from animal models and safety data from healthy individuals and are also expected to be active against mpox [22].

CDV and BDV

CDV is an intravenous drug against mpox that prevents viral replication by inhibiting DNA polymerase and DNA polymerase 3′-5′exonuclease [16,19,22,23]. BDV is an oral lipid analogue of CDV with proven efficacy in the treatment of orthopoxvirus infections in animal studies [19]. BDV is converted into the active form intracellularly through phosphorylation into CDV diphosphate (CDV-pp) and has a higher in vitro potency than CDV [19].

The major concern with CDV is its dose-dependent nephrotoxicity [19,22]. To curb this adverse effect oral probenecid is added, which prevents the uptake of CDV from the proximal renal tubules, along with intravenous normal saline pre-CDV administration with or without additional fluids post-CDV administration [19,22]. Other adverse effects of CDV include proteinuria, neutropenia, recurrent infections, iritis, uveitis, hypotony of the eye, and a decrease in serum bicarbonate [19,22,24]. CDV is contraindicated in patients with serum creatinine levels above 1.5 mg/dL, creatinine clearance of less than or equal to 55 mL/min, and urine proteins greater than or equal to 100 mg/dL (because of a risk of proteinuria) [19,24]. CDV cannot be used in patients who have used nephrotoxic agents within seven days [24]. Direct intraocular injections of CDV cannot be given due to the risk of ocular adverse effects [24]. Furthermore, CDV has shown embryotoxicity in various animal studies; as such, CDC has recommended against using CDV in pregnant or lactating females. The recent development of CDV-resistant strains of MPXV raises concerns and may lead to cross-resistance against BDV; however, the prevalence of such resistance remains unknown [19,25]. Similar to CDV, BDV has shown embryotoxicity in rats and rabbit models; therefore, it too should be avoided in pregnant and lactating women [19]. These limitations hinder the treatment of mpox, underscoring the need for improved intervention with safer alternatives.

Tecovirimat

Tecovirimat (ST-246, or TPOXX; 4-trifluoromethyl-N-(3,3a,4,4a,5,5a,6,6a-octahydro-1,3-dioxo-4,6-ethenocycloprop[f]isoindol-2-(1H)-yl)carboxamide) was identified and co-developed by SIGA Technologies (New York, USA) and the US government following extensive screening of drugs against the variola virus [19,22,26-28]. Tecovirimat is active against orthopoxviruses both in vitro and in vivo, including vaccinia, camelpox, cowpox, mousepox, variola, and mpox viruses [16]. In July 2018, it was approved by the USFDA for the management of human smallpox adhering to the risk of bioterrorism; an intravenous formulation of tecovirimat developed by SIGA Human BioArmour was also approved by the USFDA on May 19, 2022, by the FDA [19,29].

Tecovirimat acts by inhibiting the orthopoxvirus VP37 envelope-wrapping protein [29-35]. The detailed mechanism of action of the drug is shown in Figure 2. The median time (Tmax) taken by tecovirimat oral capsules to reach the maximum concentration post-administration is six hours (the range of Tmax is two to 24 hours). The plasma protein binding capacity and blood-to-plasma ratio of tecovirimat are 77-82% and 0.62-0.90, respectively, irrespective of the form of administration. Tecovirimat undergoes metabolism by hydrolysis of the amide bond and glucuronidation; the enzymes involved in this process are UGT1A1 and UGT1A4 (UGT; uridine diphosphate glucuronosyltransferase). It achieves its steady state within four to six days. The half-life (t½) of 600 mg oral and 200 mg intravenous tecovirimat is achieved by 21 hours and 19 hours, respectively [35]. Tecovirimat is a weak inducer of cytochrome P450 (CYP)3A4 and a weak inhibitor of CYP2C8 and CYP2C19 [32,35]. Tecovirimat may lead to an increase in serum levels of repaglinide due to CYP2C8 inhibition resulting in hypoglycemia; therefore, there is a need to monitor blood glucose levels and look out for hypoglycemic symptoms [32,35]. Tecovirimat also decreases serum midazolam levels, necessitating dose adjustment of midazolam for optimal effectiveness [32,35]. A decrease in serum levels of tacrolimus and sirolimus has been observed due to the induction of CYP3A4 by tecovirimat [32]. After getting metabolized, tecovirimat is eliminated in urine and faeces. Following a 600 mg single oral dose administration, 73% of the dose is eliminated in urine (predominantly, metabolites), while 23% is eliminated via faeces (predominantly, tecovirimat). 

Current evidence of tecovirimat

Although human randomized trials are lacking, various animal studies have established tecovirimat as an effective and well-tolerated antiviral to treat human mpox with no adverse effects [19]. Grosenbach et al., in their study involving monkeys and rabbits, demonstrated that the minimum dose of tecovirimat required to achieve 90% survival for 14 days was 10 mg/kg body weight against mpox and 40 mg/kg body weight against rabbitpox [31]. In another study conducted to assess prophylactic efficacy in subcutaneous models, tecovirimat demonstrated 100% protection against mpox when administered in doses of 100 mg/kg body weight within zero to four days of infection for 14 days [32,36]. The prophylactic effect of tecovirimat in intravenous models has been demonstrated in multiple studies [32]. A dose range of 3-300 mg provided 100% protection if given for five days post-infection [32]. Another study demonstrated 3 mg/kg to be an effective dose; however, 10 mg/kg also led to a decrease in lesion count and viremia [31-34]. Tecovirimat has been indicated for the treatment of human smallpox caused by variola virus, cowpox, and mpox (viruses of the same family, orthopoxvirus) by the EMA (n.d.) and for smallpox by the USFDA [35,36]. Currently, there is no data regarding the use of tecovirimat in pregnant women, although it did not have any embryotoxic or teratogenic reactions in animal trials [32,35]. There is neither any evidence of the presence of tecovirimat in human milk nor any reported effect of the drug on breastfed infants or milk production; however, tecovirimat was found to be present in animal milk. The USFDA recommends that in patients weighing 13 kg and more, tecovirimat oral capsules should be prescribed primarily; the intravenous route is only indicated when the patient cannot take oral capsules [35]. Tecovirimat capsules must be taken within 30 minutes after a full meal as food may significantly increase the absorption of tecovirimat, with a 45% increase of maximum plasma concentration and area under the curve 0-24 at a steady state [29,32,35]. The FDA-recommended dosage of tecovirimat capsules in adults and pediatric patients weighing at least 13 kg is given in Table 2 [35]. For patients who are unable to swallow the capsules, a mixture containing 30 mL of fluid or soft food and the drug is prepared, which must be swallowed within 30 minutes of its preparation. Injectable tecovirimat is given as a six-hour intravenous infusion. Intravenous tecovirimat is available in a single-dose clear glass vial containing 200 mg/20 mL drug solution. The FDA-recommended dosage of parenteral tecovirimat in adults and pediatric patients is given in Table 2. Patients weighing at least 13 kg should be switched over to oral capsules once oral therapy is tolerated to complete the 14-day course [35]. Injectable tecovirimat has a Tmax range of 6-6.5 hours (median Tmax is six hours) and the volume of distribution is 46%.

**Table 2:** Dosage of tecovirimat

Body weight (in kg)	Dosage
	Oral dosage for 14 days (number of capsules)
13 – <25	200 mg (one capsule) every 12 hours
25 – <40	400 mg (two capsules) every 12 hours
40 – <120	600 mg (three capsules) every 12 hours
≥120	600 mg (three capsules) every eight hours
	Intravenous dosage
3 – <35	6 mg/kg every 12 hours by intravenous infusion over six hours
35 – <120	200 mg every12 hours by intravenous infusion over six hours
≥120	300 mg every 12 hours by intravenous infusion over six hours

Tecovirimat capsules have no contraindications; however, the injectable form is contraindicated in patients with renal impairment. There is a risk of QT prolongation when tecovirimat is administered together with drugs causing QT prolongation [32,35]. Tecovirimat’s effect on human reproductive potential is still unknown, although testicular toxicity leading to reduced fertility has been noticed in male mice. In patients with renal or hepatic impairment, no dose adjustment is required while using tecovirimat capsules, but the parenteral form is contraindicated with creatinine clearance of less than 30 mL/min. The most common adverse effects seen with tecovirimat capsules are headache, vomiting, nausea, and abdominal pain, suggesting that it can be a better alternative compared to CDV or BDV in the treatment of mpox owing to the associated superior clinical outcomes, improved safety profile, and benign adverse effect profile [35]. Table 3 summarises all the studies included in this narrative review. 

**Table 3:** Details of the studies included in the narrative review

S. No.	Authors/Study	Study design	Primary findings
1.	Bass J et al., 2013 [6]	Original article	The “MPX curriculum” training programme helped in confidence in surveillance of mpox cases.
2.	Anderson MG et al., 2003 [8]	Case report	The third paediatric case of mpox reported in the USA from contact with rodents imported from Africa.
3.	Adegboye OA et al., 2022 [10]	Commentary	The global outbreak of MPXV in 27 countries and its geographical distribution of cases across Africa.
4.	Fahrni ML et al., 2022 [18]	Correspondence	The possibility of vertical transmission of MPXV which is associated with increased risk of maternal and perinatal co-morbidities and mortality.
5.	Farlow J et al., 2010 [25]	Original article	The presence and genomic location of mutations in MPXV which led to rise of CDV resistant strains of the virus.
6.	Duraffour S et al., 2015 [30]	Original article​​	Tecovirmat directly targets peripheral membrane protein F13L and acts as a broad-spectrum antiviral, thus inhibiting viruses belonging to the orthopoxvirus genus.
7.	Grosenbach DW et al., 2018 [31]	Original article	Tecovirimat 10 mg per kg body weight for fourteen days was the minimum dose required to achieve more than 90% survival in the mpox model. No adverse effects were reported with the use of tecovirimat in humans.
8.	Huggins J et al., 2009 [33]	Original article	Oral dosing with ST-426 once per day reduces viral load and number of lesions in animals infected with poxvirus disease.
9.	Berhanu A et al., 2015 [34]	Brief report	Post Exposure treatment with tecovirimat alone or in combination with ACAM2000 provided complete protection against mpox infection.

Limitations: This narrative review focuses only on the available treatment options of mpox and not on prevention options. Further systematic reviews may broaden the understanding of the scope of evidence available with all treatment options of mpox.

## Conclusions

Mpox is an emerging epidemic threatening to become a global burden, requiring urgent attention and swift treatment protocols. Compilation of currently available evidence suggests multi-system complications of mpox, necessitating early recognition and treatment. Out of the limited available choices for treatment, tecovirimat seems to be the preferred option owing to its favourable outcomes and safety profile. With the currently limited data, tecovirimat can be considered the antiviral of choice in the management of mpox. We recommend systematic reviews of tecovirimat evaluating its efficacy and safety in the treatment of mpox. This would also aid in framing guidelines for the management of this disease.
